# The 2019 n2c2/OHNLP Track on Clinical Semantic Textual Similarity: Overview

**DOI:** 10.2196/23375

**Published:** 2020-11-27

**Authors:** Yanshan Wang, Sunyang Fu, Feichen Shen, Sam Henry, Ozlem Uzuner, Hongfang Liu

**Affiliations:** 1 Department of Health Sciences Research Mayo Clinic Rochester, MN United States; 2 Information Sciences and Technology George Mason University Fairfax, VA United States

**Keywords:** natural language processing, clinical natural language processing, medical natural language processing, semantic textual similarity, ClinicalSTS, n2c2, electronic health records, challenge, shared task

## Abstract

**Background:**

Semantic textual similarity is a common task in the general English domain to assess the degree to which the underlying semantics of 2 text segments are equivalent to each other. Clinical Semantic Textual Similarity (ClinicalSTS) is the semantic textual similarity task in the clinical domain that attempts to measure the degree of semantic equivalence between 2 snippets of clinical text. Due to the frequent use of templates in the Electronic Health Record system, a large amount of redundant text exists in clinical notes, making ClinicalSTS crucial for the secondary use of clinical text in downstream clinical natural language processing applications, such as clinical text summarization, clinical semantics extraction, and clinical information retrieval.

**Objective:**

Our objective was to release ClinicalSTS data sets and to motivate natural language processing and biomedical informatics communities to tackle semantic text similarity tasks in the clinical domain.

**Methods:**

We organized the first BioCreative/OHNLP ClinicalSTS shared task in 2018 by making available a real-world ClinicalSTS data set. We continued the shared task in 2019 in collaboration with National NLP Clinical Challenges (n2c2) and the Open Health Natural Language Processing (OHNLP) consortium and organized the 2019 n2c2/OHNLP ClinicalSTS track. We released a larger ClinicalSTS data set comprising 1642 clinical sentence pairs, including 1068 pairs from the 2018 shared task and 1006 new pairs from 2 electronic health record systems, GE and Epic. We released 80% (1642/2054) of the data to participating teams to develop and fine-tune the semantic textual similarity systems and used the remaining 20% (412/2054) as blind testing to evaluate their systems. The workshop was held in conjunction with the American Medical Informatics Association 2019 Annual Symposium.

**Results:**

Of the 78 international teams that signed on to the n2c2/OHNLP ClinicalSTS shared task, 33 produced a total of 87 valid system submissions. The top 3 systems were generated by IBM Research, the National Center for Biotechnology Information, and the University of Florida, with Pearson correlations of *r*=.9010, *r*=.8967, and *r*=.8864, respectively. Most top-performing systems used state-of-the-art neural language models, such as BERT and XLNet, and state-of-the-art training schemas in deep learning, such as pretraining and fine-tuning schema, and multitask learning. Overall, the participating systems performed better on the Epic sentence pairs than on the GE sentence pairs, despite a much larger portion of the training data being GE sentence pairs.

**Conclusions:**

The 2019 n2c2/OHNLP ClinicalSTS shared task focused on computing semantic similarity for clinical text sentences generated from clinical notes in the real world. It attracted a large number of international teams. The ClinicalSTS shared task could continue to serve as a venue for researchers in natural language processing and medical informatics communities to develop and improve semantic textual similarity techniques for clinical text.

## Introduction

### Background

Semantic textual similarity (STS) is a common task in the general English domain to assess the degree to which the underlying semantics of 2 segments of text are equivalent to each other. Equivalency is usually assessed using ordinal scaled output ranging from complete semantic equivalence to complete semantic dissimilarity. Applications of STS include machine translation, summarization, text generation, question answering, short answer grading, semantic search, and dialogue and conversational systems.

Clinical Semantic Textual Similarity (ClinicalSTS) is the application of STS techniques in the clinical domain that attempts to measure the degree of semantic equivalence between 2 snippets of clinical text. Due to the wide adoption of electronic health record (EHR) systems, a vast volume of free-text EHR data has been generated [1], such as progress notes, discharge summaries, radiology reports, and pathology reports. The frequent use of copy and paste, templates, and smart phrases (eg, one can type a few characters that automatically expand to a longer phrase or template) has resulted in redundancy in clinical text. This reduces the quality of EHR data and adds to the cognitive burden of tracking complex medical records in clinical practice [2]. An analysis of 23,630 progress notes written by 460 clinicians showed that 18% of the text was manually entered, 46% was copied, and 36% was imported [3].

Studies that evaluated and measured redundancy in clinical text [2] showed that STS techniques are rarely applied in the clinical domain to reduce redundancy. ClinicalSTS can identify redundant clinical sentences, that is, semantically equivalent clinical texts, by computing the similarity score between 2 clinical snippets. Removing those redundant clinical sentences is vital to many clinical applications, such as clinical text summarization, clinical semantic information retrieval, and clinical decision support systems [4].

The STS shared task has been held annually since 2012 to encourage and support research in this area [5-10]. However, STS techniques have been rarely studied on clinical texts, and to our knowledge there are no clinical STS shared tasks. To motivate natural language processing (NLP) and biomedical informatics communities to study STS problems in the clinical domain, we organized the first ClinicalSTS challenge, the BioCreative/OHNLP ClinicalSTS shared task, in 2018 [11] to provide a venue for the evaluation of state-of-the-art algorithms and models by making available a real-world clinical note data set. The shared task attracted 4 participating teams that produced a total of 12 system submissions [12].

### Objective

In 2019, we continued the shared task as a collaboration with National NLP Clinical Challenges (n2c2) and the Open Health Natural Language Processing (OHNLP) consortium under the name n2c2/OHNLP track on ClinicalSTS [11]. Our aim was for the community to tackle STS problems in the clinical domain in a workshop at the American Medical Informatics Association 2019 Annual Symposium. In this paper, we first give an overview of the ClinicalSTS task and how we prepared the data set for the 2019 shared task differently from that in the previous year. Then, we describe the record number of participating teams and their systems. Finally, we present the results, system rankings, and future research directions for the ClinicalSTS task.

## Methods

### Task Overview

ClinicalSTS provides paired clinical text snippets for each participant. The clinical text snippets are mostly sentences extracted from clinical notes. The participating systems are asked to return a numerical score indicating the degree of semantic similarity between the 2 sentences. Performance is measured by the Pearson correlation coefficient between the predicted similarity scores and human judgments. The ClinicalSTS scores fall on an ordinal scale, ranging from 0 to 5, where 0 means that the 2 clinical text snippets are completely dissimilar (ie, no overlap in their meanings) and 5 means that the 2 snippets have complete semantic equivalence. Our previous publications [12,13] showed clinical text examples of the ordinal similarity scale. Participating systems can use real valued scores to indicate their semantic similarity prediction.

### Data Preparation

We collected the data set for the 2019 ClinicalSTS shared task from EHRs at the Mayo Clinic’s clinical data warehouse. Both the study and a waiver of informed consent were approved by the Mayo Clinic Institutional Review Board in accordance with 45 CFR 46.116 (approval no. 17–003030). Since the Mayo Clinic had completed a systemwide EHR transition across all care sites from GE Healthcare to Epic Systems Corporation, the data set in the 2019 shared task combined the data set from the 2018 shared task, which was an annotated subset of the MedSTS data set [13], and a new data set extracted from the historical GE EHR system and Epic EHR system. By combining data sets, we aimed to compare the semantics in clinical text generated from 2 different EHR systems. We did not release the EHR source information to the participating teams during the shared task.

**Figure 1:**
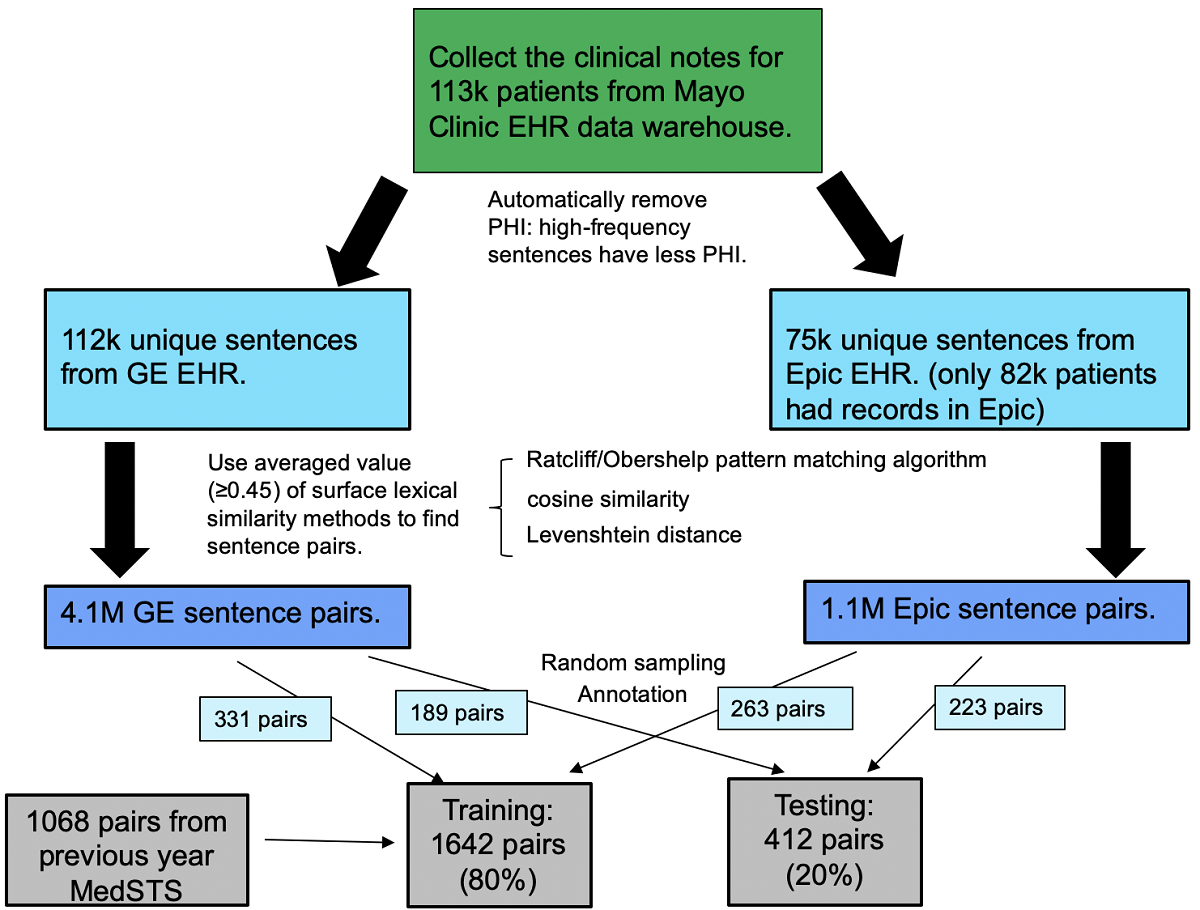
Flowchart of the released data set generation in the 2019 n2c2/OHNLP track on Clinical Semantic Textual Similarity. EHR: electronic health record; PHI: protected health information.

Figure 1 illustrates the data set used for this shared task. To curate the data set, we first collected clinical notes from the clinical data warehouse for 113,000 patients receiving their primary care at the Mayo Clinic. We removed protected health information (PHI) by employing a frequency filtering approach [14] based on the assumption that sentences appearing in multiple patients’ records tend to contain no PHI, which resulted in 112,00 unique sentences from the GE and 75,000 unique sentences from the Epic EHRs. We used the averaged value (≥0.45) of 3 surface lexical similarities, namely the Ratcliff/Obershelp pattern-matching algorithm [15], cosine similarity [16], and Levenshtein distance [17], as a cutoff value to obtain candidate sentence pairs with some level of prima facie similarity. Wang et al [13] details how these methods were employed. We obtained 4.1 million GE sentence pairs and 1.1 million Epic sentence pairs. We randomly selected 1006 sentence pairs to be annotated by human experts. To ensure that no PHI existed in the final released data set, we manually removed PHI from each sentence. In the annotation phase, we asked 2 clinical experts to independently annotate each sentence pair in the ClinicalSTS data set on the basis of their semantic equivalence. Both annotators were very knowledgeable and had many years of experience in the clinical domain. Agreement between the 2 annotators was moderate, with a weighted Cohen kappa of 0.6. We used the average of their scores as the reference standard for evaluating the submitted systems. We then randomly selected 331 GE sentence pairs and 263 Epic sentences pairs. After combining these with the previous year’s data set and removing duplicates, we finally obtained 1642 sentence pairs and released these as training data to each team to develop and fine-tune their systems. We used a total of 412 sentence pairs as the testing data set, including 189 GE sentence pairs (45.9%) and 223 Epic sentence pairs (54.1%), and asked the participating teams to return a numerical score indicating the degree of semantic similarity for each sentence pair.

### Participating Teams

Participating teams were required to sign a Data Use Agreement to get access to the challenge data set. Each team could submit up to 3 runs for the testing data, with every run having 1 line for each sentence pair that provided the similarity score assigned by the system as a floating-point number.

### Evaluation Metric

Similar to the general STS shared tasks, ClinicalSTS used the Pearson correlation coefficient between the predicted scores and the reference standard on the testing set to evaluate the submitted systems. We released a public script computing the Pearson correlation coefficient to the participating teams.

## Results

### Participating Teams

**Figure 2:**
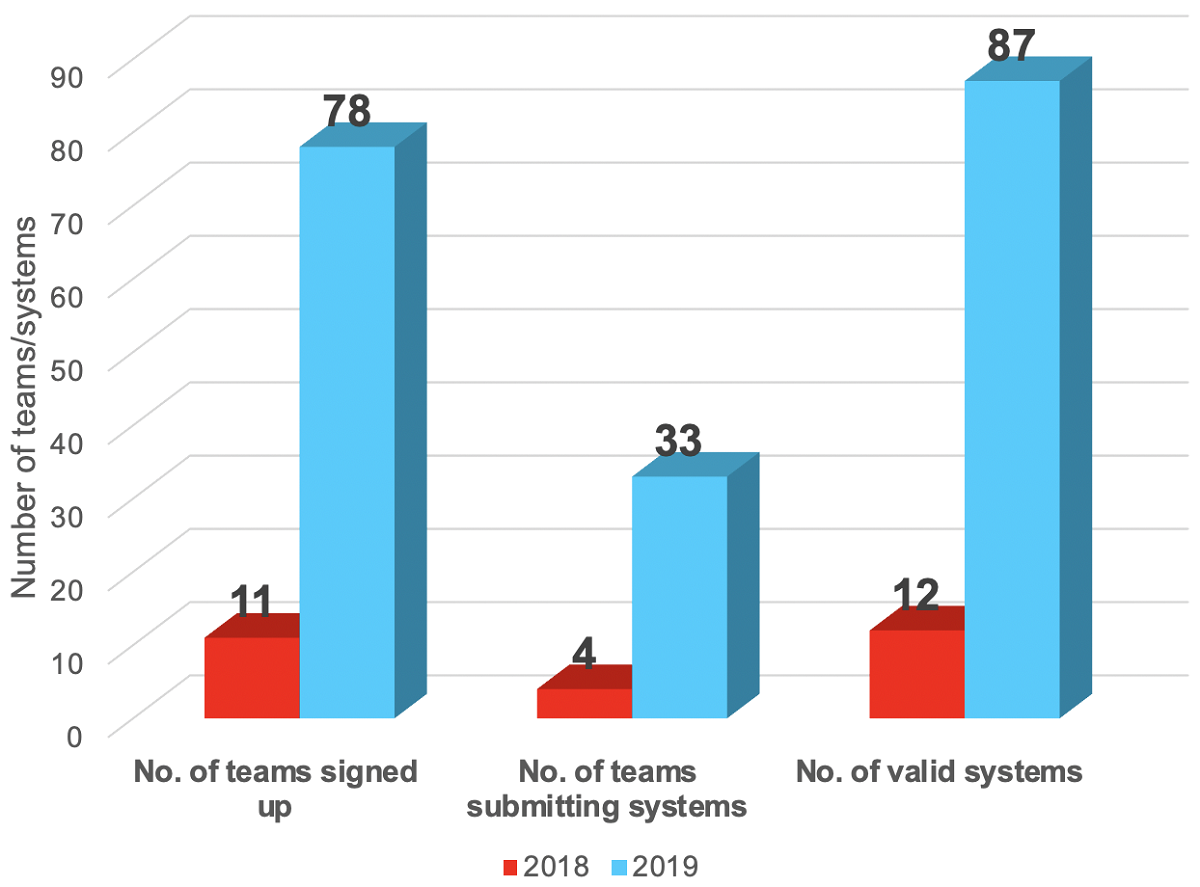
Participation in the 2019 n2c2/OHNLP Clinical Semantic Textual Similarity (ClinicalSTS) track in comparison with the 2018 BioCreative/OHNLP Clinical STS track.

Figure 2 shows the number of teams that signed up the task, teams that submitted systems, and the total number of valid systems (ie, those outputs following the submission guideline), in comparison with the 2018 BioCreative/OHNLP ClinicalSTS shared task. In summary, 78 teams from 16 countries signed up for this shared task and 33 teams submitted a total of 87 valid systems. Compared with the shared task in the previous year, the numbers of participating teams and submitted systems increased dramatically. Table 1 lists the details of teams that submitted systems, including team names, affiliations, and number of submitted systems.

**Table 1:** Participating teams, affiliations, and number of systems submitted by each.

Team name	Affiliation	Number of systems
ASU	Arizona State University, USA	3
ChangYC	National Yang-Ming University, Taiwan	3
CLEARTeamCNRSLille	N/A^a^	3
DMSS	Boston Children’s Hospital and Harvard University, USA	3
DUTIR	Dalian University of Technology, China	2
edmondzhang	Orion Health, USA	3
ezDI	ezDI Inc, USA	4
HITSZ	Harbin Institute of Technology at Shenzhen, China	3
IBMResearch	IBM Corporation, USA	0
JHU	Johns Hopkins University, USA	4
LSI_UNED	Universidad Rey Juan Carlos, Spain	3
MAH	Arizona State University, USA	3
MedDataQuest	Med Data Quest, USA	3
MICNLP	German Cancer Research Center, Germany	3
naist_sociocom	Nara Institute of Science and Technology, Japan	3
NCBI	National Center for Biotechnology Information, USA	3
nlpatvcu	Virginia Commonwealth University, George Mason University, USA	3
PUCPR	Pontifical Catholic University of Paraná, Brazil	2
QUB	Queen’s University, UK	4
SBUnlp	Stony Brook University, USA	3
superficialintelligence0405	N/A	3
UAveiro	University of Aveiro, Portugal	3
UFL	University of Florida, USA	3
UH_RiTUAL	University of Texas at Houston, USA	3
Utah-VA	University of Utah and Veterans Affairs, USA	3
vjaneja	University of Maryland, USA	1
WSU-MQ	Western Sydney University, Australia	3
Yale	Yale University, USA	3
Yuxia	University of Melbourne, Australia	2
zhouxb	Yunnan University, China	3

^a^N/A: not available.

### Basic Information of the Released Data Set

**Figure 3:**
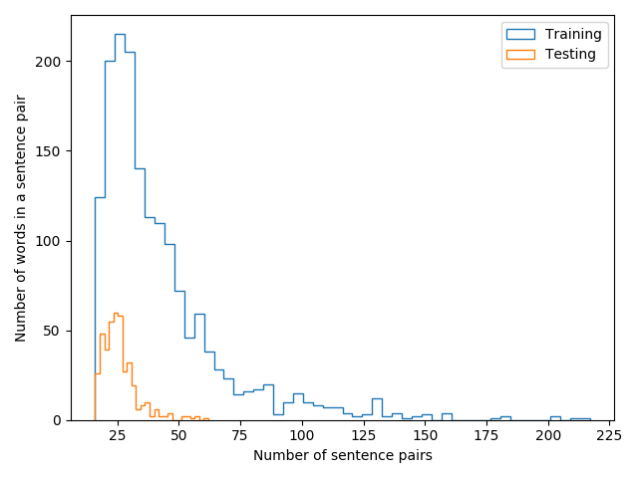
Distribution of number of words in sentence pairs in the released training and testing data sets.

Our previous publication [13] provides more detailed information about the larger MedSTS data set. We used the Python NLP package spaCy version 2.1 (ExplosionAI GmbH) tokenizer to count the total number of words in each sentence pair. Figure 3 depicts the distribution of the number of words in sentence pairs in the released training and testing data sets. Most sentences pairs had 25 to 50 words, and there were more lengthy sentences in the training data set. However, the length distribution between training and testing was consistent. Table 2 lists the number of sentence pairs with different similarity scores in the released training and testing data sets. There were more sentence pairs with similarity scores between 3 and 4 in the training data set, whereas there were more sentence pairs with similarity scores between 1 and 2 in the training data set. This might have been due to sampling bias during data set creation.

**Table 2:** Number of sentence pairs with different similarity scores in the released training and testing data sets.

Similarity score	Training data set, n	Testing data set, n
[0,1)	185	98
[1,2)	236	168
[2,3)	245	30
[3,4)	607	34
[4,5]	369	82

In addition, we used 3 surface lexical similarity methods as the baseline method to calculate the similarity scores in the testing data set, namely the Ratcliff/Obershelp pattern-matching algorithm, cosine similarity, and Levenshtein distance similarity. For more details about these baselines, please refer to our previous publication [13]. The cosine similarity achieved the best performance among the 3 baselines, with a Pearson correlation of *r*=.3709, followed by Levenshtein distance similarity with *r*=.2816 and Ratcliff/Obershelp with *r*=.2480.

### Participating System Performance and Rankings

Table 3 lists the overall performance of all the valid submitted systems and the comparison with overall performance in the previous year’s challenge. Table 4 shows the top 10 teams with their specific corresponding best runs and performance. The best system was from the team IBM Research’s LM-POSTPROCESS-RUN with a Pearson correlation coefficient of *r*=.9010, an 8.2% increase from the previous year’s best system. Overall, the median correlation score for the testing data set was *r*=.8291, a 3.4% increase from the previous year. We also compared the best run with other top systems using the Wilcoxon signed rank *t* test (Table 4). We found no statistically significant difference in 9 out of the top 10 systems (*P*<.001).

**Table 3:** Overall performance of the valid submitted systems and comparison with the previous year’s results.

Metric	2019 n2c2/OHNLP ClinicalSTS^a^, *r*	2018 BioCreative/OHNLP ClinicalSTS, *r*
Maximum	.9010	.8328
Minimum	–.0530	.7005
Median	.8291	.8016
Mean	.7183	.7820
Standard deviation	.2260	.0476

^a^ClinicalSTS: Clinical Semantic Textual Similarity.

**Table 4:** Performance of the top 10 teams with the corresponding best runs.

Rank	Team	Run	*r*	*P* value
1	IBMResearch	LM-POSTPROCESS-RUN	.9010	—^a^
2	NCBI	1	.8967	.88
3	UFL	XLNet-Run	.8864	.40
4	DMSS	AVERAGE-Run	.8792	.45
5	Yale	3	.8784	.09
6	QUB	fine_tuned_models_mean-Run	.8704	.54
7	MICNLP	Step1	.8694	<.001
8	HITSZ	raw_ensemble	.8685	.80
9	SBUnlp	ensembleall	.8677	.003
10	JHU	BERT-w-stsb-run	.8543	.005

^a^Not applicable.

We also compared the performance of valid systems for sentence pairs from GE and Epic EHR systems in the testing data set (Table 5). Overall, the participating systems performed better on the Epic sentence pairs than on the GE sentence pairs, despite the fact that a much larger portion of the training data were GE sentence pairs. This result indicates that the clinical sentences in our data set collected from the Epic EHR might be semantically simpler than those collected from the GE EHR system, which makes it easier for machine or deep learning models to learn the sentence semantic meaning.

**Table 5:** Performance comparison (Pearson correlation coefficient) between the Epic and GE sentence pairs.

Metric	Epic (n=223), *r*	GE (n=189), *r*
Maximum	.9148	.9022
Minimum	.0917	.0070
Median	.8377	.7785
Mean	.7792	.6812
Standard deviation	.1649	.2257

Table 6 shows the top 5 systems for the Epic and GE sentence pairs. The system from IBM Research achieved the best performance for the GE sentence pairs, which is consistent with their overall performance. Yale University’s system (Run 4) had the best performance for the Epic sentence pairs, while the same system was not even in the top 5 performing systems for the GE sentence pairs.

**Table 6:** Top 5 systems for sentence pairs from the Epic and GE electronic health record systems.

Rank		Team	Run	*r*
**Epic**				
	1	Yale	4	.9148
	2	IBMResearch	LM-POSTPROCESS-RUN	.9098
	3	NCBI	1	.9020
	4	DMSS	AVERAGE-Run	.8949
	5	UFL	Assemble-Run	.8863
**GE**				
	1	IBMResearch	LM-POSTPROCESS-RUN	.9022
	2	UFL	XLNet-Run	.9010
	3	NCBI	1	.8938
	4	Yale	3	.8796
	5	MICNLP	Step1	.8576

### Methods Used in the Participating Systems

Table 7 briefly summarizes the techniques used by the top teams. Most teams used state-of-the-art NLP neural language models in their systems, such as BERT [18] and XLNet [19], and state-of-the-art training schemas in deep learning, such as pretraining and fine-tuning schema, and multitask learning [20]. The outcomes from the top performing systems showed the advantages of these techniques over conventional machine learning and language models in learning semantics in human language, particularly in clinical language. Having said that, given the nature of the semantic simplicity of the sentences in the ClinicalSTS data set, neural language models and these training schemas need further comprehensive evaluation on larger clinical corpora with more complex sentences and semantics.

**Table 7:** Brief summary of the techniques used in the top systems.

Team	Techniques
IBMResearch	Multitask learning, BioBERT, RoBert, ClinicalBERT
NCBI	Convolutional neural network, multitask learning, BERT
UFL	BERT, XLNet
DMSS	BERT, XLNet
Yale	BERT, graph convolutional neural network
QUB	BERT, XLNet
MICNLP	BERT, medication graph
HITSZ	BERT, cTAKES
SBUnlp	BERT, Unified Medical Language System
JHU	BERT
Utah-VA	Multiple natural language processing features, deep neural network

## Discussion

### Principal Findings

We have given an overview of the 2019 n2c2/OHNLP ClinicalSTS shared task that aimed to measure the degree of semantic equivalence between 2 snippets of clinical text. We described how we prepared the data set in this year’s shared task differently from that in the previous year, the participating teams and their systems, and the results. We witnessed an increasing research interest in the ClinicalSTS task among the NLP and medical informatics communities and increased system performance for the task. We also observed several limitations during the data preparation. There were limitations in the reference standard data creation, particularly for annotating the medication-related sentence pairs in the data set. Concerns were raised by participating teams regarding the judgement for those pairs. Table 8 shows an example of such a sentence pair. One may question that why the minocycline-oxycodone pair should have a much higher score than the oxycodone-pantoprazole pair. Minocycline is an antibiotic, and pantoprazole is an antacid. One annotator mentioned that the score of oxycodone + antibiotics was greater than the oxycodone + antacid score based on his experience of seeing them more frequently in the EHRs. In addition, the first case mentioned taking minocycline daily, whereas the second case did not mention that pantoprazole should be taken once daily (such semantic information is missing in this case). Two of the annotators were nurses with a medical background but were not pharmacists. Both annotators agreed that in future work, involving pharmacists to annotate drug sentences could help make the annotation more accurate because drug sentences should be scored based on drug mechanisms, indications, doses, application period, and disease stages, plus pharmacogenomics and epigenomics or proteomics, etc.

**Table 8:** Examples of medication-related sentence pairs in the data set.

Examples	Score
sentence1: minocycline [MINOCIN] 100 mg capsule 1 capsule by mouth one time daily. sentence2: oxycodone [ROXICODONE] 5 mg tablet 1-2 tablets by mouth every 4 hours as needed.	3.0
sentence1: oxycodone [ROXICODONE] 5 mg tablet 0.5-1 tablets by mouth every 4 hours as needed. sentence2: pantoprazole [PROTONIX] 40 mg tablet enteric coated 1 tablet by mouth Bid before meals.	1.0

We also found that some sentence pairs seemed to be semantically equivalent but were assigned low similarity scores. For example, sentence 1 is “Thank you for choosing the Name, APRN, C.N.P., M.S. care team for your health care needs!” and sentence 2 is “Thank you for choosing the Name, M.D. care team for your health care needs!” The reason for the score (4.0) is that the degree of the provider is different. The provider in the first sentence is a nurse, whereas that in the second sentence is a physician. Thus, these 2 sentences are not equivalent. Another example is sentence 1: “Thank you for choosing the Name M.D. care team for your health care needs!” and sentence 2: “Thank you for allowing us to assist in the care of your patient.” The reason for the score (2.0) is that the first sentence contains more details about the provider, whereas the second has fewer details.

Although there was a record number of 87 valid systems participating in the shared task, this is still not large enough to be able to extrapolate statistical analysis results to draw a convincing conclusion. The performance difference of these participating systems in the sentence pairs from different EHR systems may be attributable to bias in the system and the sampling data set.

In our future work, we might subcategorize the sentence pairs into different topics, such as medication or clinical workflow. We could provide tailored annotation guidelines according to the topic and invite subdomain experts with specific background (eg, pharmacist) to review sentences pairs in different topics (eg, medication-related sentence pairs).

### Conclusions

ClinicalSTS is an important technique in many downstream clinical applications, such as clinical text summarization, clinical semantic information retrieval, and clinical decision support systems. In this paper, we provided an overview of the 2019 n2c2/OHNLP ClinicalSTS shared task that focused on computing semantic similarity for clinical text sentences generated from clinical notes in the real world. For this shared task, 33 international teams submitted a total of 87 valid systems. The top performing systems applied state-of-the-art NLP neural language models, such as BERT and XLNet, and state-of-the-art training schemas in deep learning, such as pretraining and fine-tuning schema. The best system used multitask learning and achieved a Pearson correlation coefficient of *r*=.9010, an 8.2% increase from the previous year’s best system. We also compared the performance for sentences from both GE and Epic EHR systems and found better performance on the Epic sentence pairs than on the GE sentence pairs. The ClinicalSTS task remains challenging given the complexity of clinical texts. The ClinicalSTS shared task could continue to serve as a venue for researchers in NLP and medical informatics communities to develop and improve STS techniques for clinical text.

## Abbreviations

ClinicalSTS: Clinical Semantic Textual Similarity
EHR: electronic health record
n2c2: National NLP Clinical Challenges
NLP: natural language processing
OHNLP: Open Health Natural Language Processing
PHI: protected health information
STS: semantic textual similarity
